# Supplementary material for: MetaRibo-Seq measures translation in microbiomes
Source: Nat Commun. 2020 Jun 29;11:3268. doi: 10.1038/s41467-020-17081-z (PMC7324362; doi:10.1038/s41467-020-17081-z)
Supplement: Supplementary file 10 — Supplementary Data 7 [file 41467_2020_17081_MOESM10_ESM.zip › File2/Confidence_High_Taxonomy/275483_out.krona.html]

Javascript must be enabled to view this page.

members
magnitude
magnitudeUnassigned
count
unassigned
taxon
rank

275483\_out

53

2
superkingdom
53

53
1239
phylum

class
186801
53

186802
order
53

541000
family
53


SRS055017\_contig\_number\_contig-100\_23830.74456SRS104693\_contig\_number\_contig-100\_40332.40333
1263
genus
53
2


SRS101433\_contig\_number\_9667SRS142890\_contig\_number\_19750
species
2293194
2

38

SRS011084\_contig\_number\_22866SRS011405\_contig\_number\_14564SRS014855\_contig\_number\_2499SRS014979\_contig\_number\_13175SRS015578\_contig\_number\_3139SRS015782\_contig\_number\_42036SRS016954\_contig\_number\_27130SRS018133\_contig\_number\_14535SRS018351\_contig\_number\_13299SRS019030\_contig\_number\_16157SRS020394\_contig\_number\_5697SRS020869\_contig\_number\_10236SRS022071\_contig\_number\_29513SRS022524\_contig\_number\_376SRS024625\_contig\_number\_17172SRS024663\_contig\_number\_4988SRS042628\_contig\_number\_16367SRS050998\_contig\_number\_15276SRS063040\_contig\_number\_30224SRS065504\_contig\_number\_contig-100\_962.200635SRS076804\_contig\_number\_17777SRS077294\_contig\_number\_13374SRS098061\_contig\_number\_10145SRS098644\_contig\_number\_33270SRS1041033\_contig\_number\_contig-100\_30973.64568SRS1055049\_contig\_number\_14638SRS142980\_contig\_number\_3021SRS143372\_contig\_number\_7925SRS143523\_contig\_number\_18213SRS144183\_contig\_number\_4830SRS144362\_contig\_number\_26068SRS147425\_contig\_number\_10270SRS148319\_contig\_number\_2895SRS893259\_contig\_number\_5202SRS893288\_contig\_number\_156SRS893292\_contig\_number\_8842SRS893295\_contig\_number\_12077SRS893300\_contig\_number\_11657
species
41978

165186
species

SRS016495\_contig\_number\_16926SRS017103\_contig\_number\_40384SRS018541\_contig\_number\_12062SRS018817\_contig\_number\_12798SRS045826\_contig\_number\_7135SRS893270\_contig\_number\_14840
6

4

SRS024331\_contig\_number\_33563SRS047014\_contig\_number\_34036SRS140513\_contig\_number\_contig-100\_24843.63408SRS971276\_contig\_number\_13987
species
1160721

2293177
species

SRS149181\_contig\_number\_22333
1
